# β-catenin/Wnt signaling controls progenitor fate in the developing and regenerating zebrafish retina

**DOI:** 10.1186/1749-8104-7-30

**Published:** 2012-08-24

**Authors:** Jason R Meyers, Lily Hu, Ariel Moses, Kavon Kaboli, Annemarie Papandrea, Pamela A Raymond

**Affiliations:** 1 Department of Biology, Colgate University, Hamilton, NY, 13346, USA; 2 Molecular, Cellular and Developmental Biology, University of Michigan, Ann Arbor, MI, 48104, USA

**Keywords:** β-catenin/Wnt, 1-azakenpaullone, XAV939, Retina, Development, Regeneration, Proliferation, Zebrafish, Ciliary marginal zone

## Abstract

**Background:**

The zebrafish retina maintains two populations of stem cells: first, the germinal zone or ciliary marginal zone (CMZ) contains multipotent retinal progenitors that add cells to the retinal periphery as the fish continue to grow; second, radial glia (Müller cells) occasionally divide asymmetrically to generate committed progenitors that differentiate into rod photoreceptors, which are added interstitially throughout the retina with growth. Retinal injury stimulates Müller glia to dedifferentiate, re-enter the cell cycle, and generate multipotent retinal progenitors similar to those in the CMZ to replace missing neurons. The specific signals that maintain these two distinct populations of endogenous retinal stem cells are not understood.

**Results:**

We used genetic and pharmacological manipulation of the β-catenin/Wnt signaling pathway to show that it is required to maintain proliferation in the CMZ and that hyperstimulation of β-catenin/Wnt signaling inhibits normal retinal differentiation and expands the population of proliferative retinal progenitors. To test whether similar effects occur during regeneration, we developed a method for making rapid, selective photoreceptor ablations in larval zebrafish with intense light. We found that dephosphorylated β-catenin accumulates in Müller glia as they re-enter the cell cycle following injury, but not in Müller glia that remain quiescent. Activation of Wnt signaling is required for regenerative proliferation, and hyperstimulation results in loss of Müller glia from the INL as all proliferative cells move into the ONL.

**Conclusions:**

β-catenin/Wnt signaling is thus required for the maintenance of retinal progenitors during both initial development and lesion-induced regeneration, and is sufficient to prevent differentiation of those progenitors and maintain them in a proliferative state. This suggests that the β-catenin/Wnt cascade is part of the shared molecular circuitry that maintains retinal stem cells for both homeostatic growth and epimorphic regeneration.

## Background

Adult teleost fish have retinal stem cells that mediate both continued growth of the neural retina and regeneration following injury. The fish retina increases in size from a proliferating neuroepithelium residing at the perimeter of the neural retina adjacent to the ciliary epithelium, termed the ciliary marginal zone (CMZ) [1,2]. These stem cells produce multipotent progenitors capable of differentiating into all retinal cell fates. In response to damage, the normally quiescent Müller glial cells within the differentiated central regions of the teleost retina dedifferentiate and reenter the cell cycle to produce multipotent progenitors that replace the lost cells [2-6]. It is not clear whether the cell-cell signals that transiently maintain the regenerative stem cell pool following injury are the same as those involved in maintaining the permanent stem cell compartment in the CMZ.

β-catenin/Wnt signaling has been identified in the control of stem cell fate decisions in a variety of tissues including skin, neural stem cells, and intestinal crypts [7,8]. Studies in chickens, *Xenopus*, and zebrafish suggest that Wnt may play a similar role in maintaining retinal progenitors during development [9-16]. Wnt signaling has also been implicated in the limited, non-regenerative, Müller-glial proliferation that occurs following injury in the murine retina [17,18], and was recently suggested to be necessary and sufficient for Müller glial proliferation in the regenerating adult zebrafish retina [19].

Since the zebrafish retina has both an active population of stem cells in the CMZ, and a robust regenerative proliferation of the Müller glial cells, it is an ideal system in which to test the roles of β-catenin/Wnt signaling in the control of these two distinct populations of retinal progenitors. Repression of Wnt signaling is required for initial anterior specification, and *masterblind* (*mbl*)/*axin1* mutant zebrafish that have constitutively hyperactive Wnt signaling lack or have greatly reduced forebrain and retina [20-22]. We show that, following gastrulation, genetic or pharmacological activation of the β-catenin/Wnt cascade in zebrafish embryos blocks retinal differentiation and promotes a retinal progenitor fate, while inhibition of the β-catenin/Wnt cascade results in loss of the proliferative neuroepithelium in the CMZ. In response to light-induced loss of photoreceptors in larval zebrafish, β-catenin is stabilized in Müller glia as they re-enter the cell cycle. Inhibition of Wnt-signaling after light damage prevents the injury-induced activation of Müller glia, while constitutive activation of the cascade biases activated Müller glia to remain as proliferative progenitors, resulting in the loss of differentiated Müller glia from the INL. Together these data suggest that Wnt signaling is a key regulator of retinal stem cell fate in both the CMZ niche and in the injury-induced dedifferentiation of Müller glia into a regenerative stem cell.

## Results

### Early hyperactivation of Wnt signaling prevents eye-field formation

**Figure 1:**
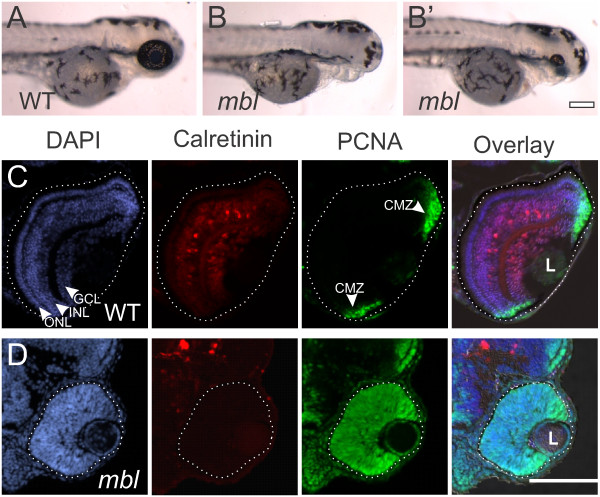
**Small retinas in *masterblind *fish fail to differentiate.** Compared with wild-type (WT) fish at 72 hours post fertilization (hpf) **(A)**, *masterblind* (*mbl*) fish typically lack forebrain and eyes **(B)**, although some fish develop with a small eye **(B’)**. Cryosections of wild-type retinas at 72 hpf **(C)** reveal lamination of the nuclei, labeled with DAPI in blue, into three layers: the ganglion cell layer (GCL) nearest the lens (L), the inner nuclear layer (INL), and the outer nuclear layer (ONL). Calretinin-labeled neurons have differentiated in the GCL and INL. Proliferative cells, marked with proliferating cell nuclear antigen (PCNA) are found only in the periphery of the retina in the ciliary marginal zone (CMZ). In contrast, cryosections of *mbl* retinas at 72 hpf **(D)** show a lack of nuclear lamination, a failure of calretinin-positive neurons to differentiate, and all cells within the retina remain PCNA-positive. In these and all following figures, the eye is outlined with a dotted line to distinguish it from adjacent tissues. Scale bar: A, B, 200 μm; C, D, 100 μm.

Wnt signaling is hyperactive in zebrafish that contain a mutation in the *axin1* gene (*masterblind mbl;*[21]). As Wnt signaling is constitutively active, and suppression of Wnt signaling is required for anterior development, these fish fail to develop forebrain structures and eyes are absent or greatly reduced (Figure 1A, B) [21]. The *mbl* mutation demonstrates incomplete penetrance, and some females produced offspring that instead of having no eyes develop small eyes (Figure 1B’). Cryosections of wild-type retinas at 72 hours post-fertilization (hpf) show normal lamination into the three nuclear layers: the ganglion cell layer (GCL) closest to the lens, the inner nuclear layer (INL), and the outer nuclear layer (ONL) at the back of the retina (Figure 1C). Calretinin-positive neurons have differentiated in the GCL and INL and the only proliferative cells (marked by expression of proliferating cell nuclear antigen (PCNA)) are at the periphery of the retina in the CMZ (Figure 1C). Thus at 72 hpf, initial retinal differentiation is complete. However, in the small-eye *mbl* retinas at 72 hpf, all cells within the retina remained PCNA-positive, indicating that they were still in the cell cycle, failed to laminate into the three nuclear layers, and showed no expression of photoreceptor (not shown) or neural markers (Figure 1D). This suggests that Wnt signaling must be repressed for normal differentiation of the neural retina. However, since these fish have constitutively hyperactive Wnt signaling, it is difficult to separate the early versus the later effects of hyperactive Wnt signaling on retinogenesis.

**Figure 2:**
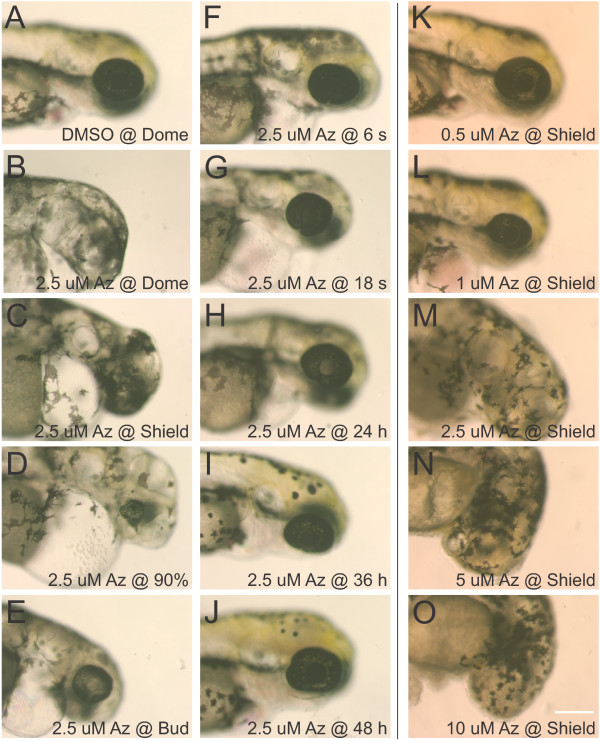
**Dose and timing-response for 1-azakenpaullone on retinal development.** The GSK3β inhibitor 1-azakenpaullone was applied to embryos at various stages of development and observations were made at 72 hpf. While control embryos treated with 0.1% DMSO as early as dome stage developed normally **(A)**, treatment with 2.5 μM 1-azakenpaullone (Az) at dome or shield stage resulted in loss of forebrain and eyes **(B, C)**. Treatment with 2.5 μM 1-azakenpaullone at 90% epiboly resulted in a small eye lacking a lens **(D)**. At bud-stage or later, treatment with 1-azakenpaullone led to a small eye, with the size of the eye increasing with progressively later treatments **(E-J)**. The effects of 1-azakenpaullone are dose-dependent: embryos treated at shield stage with 0.5 μM 1-azakenpaullone have relatively normal development **(K)**, 1 μM results in a small eye **(L)**, and treatments at 2.5 μM or higher results in failure of eye development **(M-O)**. Scale bar = 200 μm.

In order to more precisely control Wnt signaling, we used pharmacological manipulation, which allows both the level of inhibition and the timing of that inhibition to be manipulated. Both lithium and 1-azakenpaullone inhibit the activity of GSK3β, mimicking activation of Wnt signaling; lithium replaces a required magnesium ion [23,24], while 1-azakenpaullone acts as an ATP-competitive inhibitor [25]. To test the temporal requirements for Wnt signaling, we applied 2.5 μM azakenpaullone to zebrafish embryos at shield, 90% epiboly, 6 somite, 18 somite, or 24 somite stages, and embryos were maintained in the drug until 72 hpf. When 1-azakenpaullone was applied at or prior to 90% epiboly, zebrafish developed without eyes (Figure 2B, C). When 1-azakenpaullone was applied after 90% epiboly, the fish developed with small eyes (Figure 2D-J). Similar results were obtained using 0.3 M LiCl for 1 h at shield or 24 hpf (data not shown). We also examined the dose response, treating embryos in 0.5 μM, 1 μM, 2.5 μM, 5 μM, and 10 μM 1-azakenpaullone at shield stage (Figure 2K-O). Fish treated with 2.5 μM or more 1-azakenpaullone developed without an eye, while embryos treated with 1 μM or less developed a small eye.

### Hyperactivation of Wnt signaling during retinogenesis blocks retinal differentiation and expands the progenitor population

**Figure 3:**
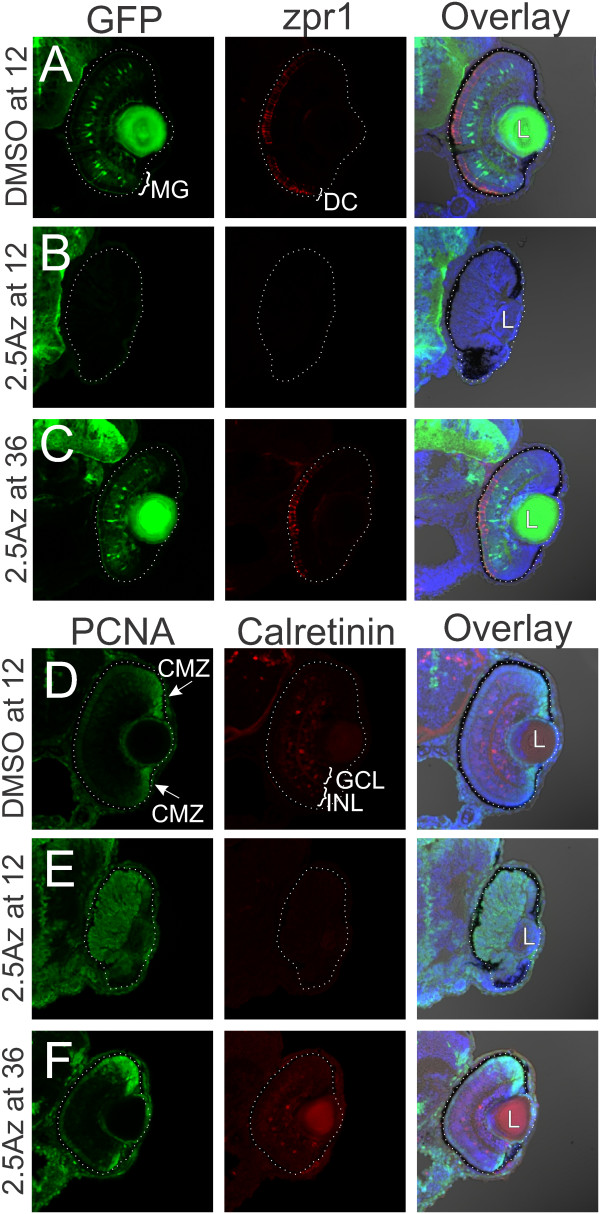
**Inhibition of GSK3β blocks retinal differentiation and maintains proliferative progenitors.***Tg*(*gfap:GFP*)^*mi2002*^ zebrafish embryos were treated with 0.025% DMSO as a vehicle control or 2.5 μM 1-azakenpaullone to inhibit GSK3β at various times during development. Retinal cryosections from DMSO-treated embryos show normal development of Müller glia (MG; GFP under control of a glial-specific promoter), photoreceptors (the zpr-1 antibody marks red-green double cones (DC)), and calretinin-positive neurons in the ganglion cell layer (GCL) and INL **(A, D)**. The fluorescent signal in the lens (L) is non-specific. Proliferative cells marked with PCNA are found at the retinal margins in the CMZ **(D)**. In contrast, retinas from embryos treated with 1-azakenpaullone from 12 hpf show no expression of GFAP, Zpr-1 labeled photoreceptors, or calretinin **(B, E)**. Nuclear lamination is absent in these retinas, and proliferative cells labeled with PCNA extend throughout the retina **(E)**. Treatment from 36 hpf allows more differentiation to occur, and the central retina is laminated **(C)**. However, the CMZ, which contains proliferative cells, is expanded **(F)**. Scale bar = 100 μm.

In order to test how activation of Wnt signaling affected retinogenesis after initial eye-field specification was complete, we treated transgenic embryos expressing GFP under control of the *gfap* promoter [26] with the GSK3β inhibitor 1-azakenpaullone beginning at 12 hpf (approximately 6 somite), prior to any retinal differentiation, at 24 hpf, when differentiation of the retina is just beginning [27], and at 36 hpf continuing until 72 hpf when initial retinogenesis is largely complete. By 72 hpf, control retinas treated with 0.025% DMSO have fully laminated into three nuclear layers (Figure 3A, D). To assess retinal cell differentiation, we used cell specific transgenic and immunocytochemical markers. In the transgenic zebrafish line Tg (*gfap:gfp*)^mi2002^, the GFP reporter is under control of the astroglial-specific promoter, glial fibrillary acidic protein, and is expressed in differentiated Müller glia*,* whose nuclei are located in the INL (Figure 3A). Photoreceptor nuclei are in the ONL; red-green double cones are labeled with the specific marker zpr-1 (Figure 3A), and rods are labeled with zpr-3, (Additional file 1: Figure S1A). Calretinin-positive retinal neurons are found in both the ganglion cell layer (GCL) and INL (Figure 3D). At 72 hpf, PCNA-positive proliferative cells are restricted to the ciliary marginal zone (CMZ) at the margin of the retina (Figure 3D). To confirm that the PCNA + cells were actively progressing through the cell cycle, fish were treated with 10 mM BrdU 2 h prior to fixation; the only cells incorporating BrdU, indicating passage through S-phase, are in the CMZ at the retinal margin (Additional file 1: Figure S1A).

When embryos were treated with 1-azakenpaullone at 12 hpf, prior to retinal differentiation, we found no expression of any markers of differentiated retinal cells, and all cells in the retinal primoridum were PCNA-positive (Figure 3B, E), and could also be labeled with a pulse of BrdU (Additional file 1: Figure S1B). Thus retinal differentiation was completely blocked by activation of Wnt signaling by inhibition of GSK3β. Retinas from embryos treated with 1-azakenpaullone at 24 hpf (Additional file 1: Figure S1C, E, F) or 36 hpf, after retinal differentiation has commenced, showed some lamination and expression of markers of retinal differentiation in central retina, particularly at 36 hpf, although there are enlarged zones of PCNA-positive and BrdU-positive cells at the retinal margins, suggesting expansion of the CMZ at the expense of normal differentiation (Figure 3C, Additional file 1: Figure S1D). Treatment with 0.3 M LiCl, which also inhibits GSK3β [23], for 1 h at 48 hpf gave a similar result when examined at 96 hpf; an expanded proliferative zone at the margins of the retina compared to fish treated with 0.3 M NaCl as a control, and maintenance of scattered proliferative cells in the INL and ONL even in the regions where some differentiation was noted (Additional file 1: Figure S1G, H).

We quantified the fraction of each retinal section that was comprised of proliferative (PCNA-positive) cells at 72 hpf. In control, DMSO-treated retinal sections, the proliferative cells at the retinal margin comprised 11.6% ± 0.7% of the area of each section at 72hpf. In contrast, for fish treated with 1-azakenpaullone beginning at 24 hpf, the proliferative cells extended across the retina comprising 73.9% ± 2.6% of the area of each section, and for fish treated with 1-azakenpaullone beginning at 36 hpf, the proliferative cells occupied 24.2% ± 1.2% of the area of each section (*n* = 10 retinas for each condition). Thus, 1-azakenpaullone maintains retinal progenitors, with early treatment maintaining the majority of cells in a proliferative state and later treatment increasing the fraction of proliferative progenitors at the retinal periphery. The difference in overall size may also reflect changes in the rate of cell cycle, as it has been previously suggested that Wnt signaling may slow the rate of cycling progenitors [14].

Notably, although activation of Wnt signaling in GSK3β-inhibited retinas retains cells in a proliferative state, the retinas of 1-azakenpaullone-treated fish are somewhat smaller than control retinas. One important reason for this may be changes in cell size, as proliferative stem and progenitor cells typically have very little cytoplasm, while differentiating neurons elaborate extensive dendritic and axonal arbors, and photoreceptors begin to develop outer segments. To determine whether this could, at least in part, explain the difference in size between the control and GSK3β-inhibited retinas, we measured the cell density in a 30 μm arc of central retina for control retinas and retinas treated with 1-azakenpaullone-treated from 24 to 72 hpf. We counted the number of nuclei across the retina within this region and measured the total area encompassed by those cells to calculate a cell density, which we then normalized to the number of cells present in 1,000 μm^2^ of retina. While control fish had 31.6 ± 1.1 cells per 1,000 μm^2^ of retina, fish treated with 1-azakenpaullone had 41.2 ± 1.4 cells per 1,000 μm^2^ of retina, a significant increase in the density (*P* < 0.001; *t*-test; *n* = 7 retinas). Thus, the differentiating cells are much less densely packed than the undifferentiated cells in GSK3β-inhibited retinas, and part of the difference in overall size of the retinas may be due to this difference in density as cells in control retinas elaborate their extensive processes as they differentiate.

**Figure 4:**
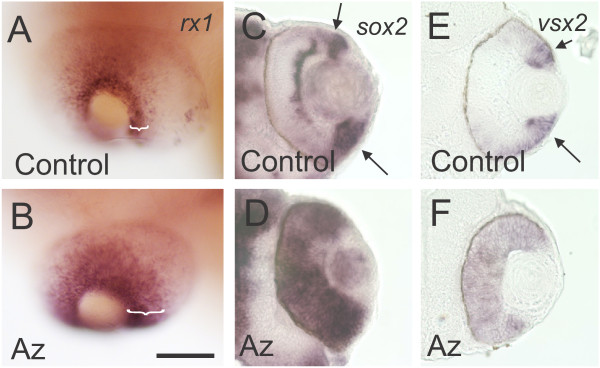
**Inhibition of GSK3β leads to expansion of retinal progenitor markers. (A, B)***In situ* hybridization with an *rx1* riboprobe. Control zebrafish embryos at 72 hpf express *rx1* in the CMZ at the retinal margin (**A**; bracket). In contrast, embryos treated with 1-azakenpaullone from 24 hpf to 72 hpf show a marked expansion in the domain of expression of *rx1*, marking an expanded CMZ (**B**; bracket). **(C, D)** Sections through retinas labeled via *in situ* hybridization with a *sox2* riboprobe. Control retinas show *sox2* expression within the CMZ (arrows) and in developing amacrine cells **(C)**. 1-azakenpaullone-treated retinas have *sox2* expression throughout the retina **(D)**. **(E, F)** Sections through retinas labeled via *in situ* hybridization with a *vsx2* riboprobe. Control retinas show *vsx2* expression within the CMZ (**E**; arrows). 1-azakenpaullone-treated retinas have *vsx2* expression throughout the retina **(F)**.

To test whether the cells that fail to differentiate in the GSK3β-inhibited retinas maintain their identity as retinal progenitor cells, we assayed expression of the retinal homeobox gene, *rx1*, in control and GSK-inhibited retinas. At 72 hpf, *rx1* is normally expressed in the progenitor cells of the CMZ (Figure 4A), but in retinas treated with 1-azakenpaullone from 24 to 72 hpf the zone of *rx1*-expression in the CMZ is expanded (Figure 4B), similar to the expansion of proliferative cells (Figure 3F). We also found that pharmacological activation of Wnt signaling leads to expanded expression of both *sox2* and v*sx2*/*Chx10* (Figure 4D, F), transcription factors associated with retinal progenitors and expressed in the CMZ of control fish (Figure 4C, E). Together, these data suggest that stimulation of Wnt signaling leads to an expansion of proliferative progenitors at the expense of normal retinal differentiation, and that these progenitors retain their retinal identity.

**Figure 5:**
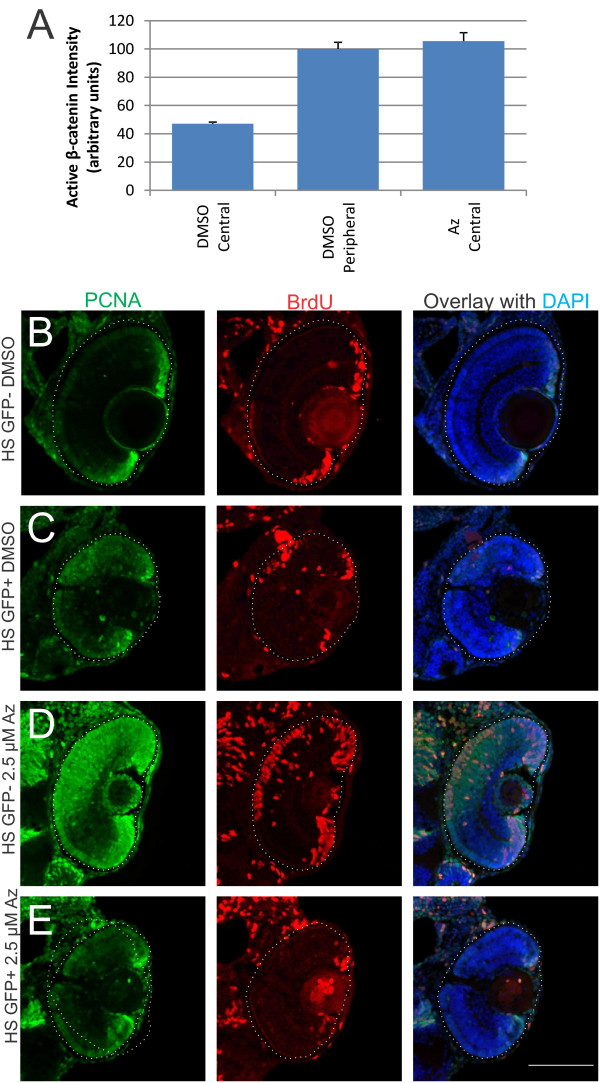
**Effects of 1-azakenpaullone are mediated via Wnt/β-catenin pathway. (A)** Quantification of fluorescent intensity from an anti-dephosphorylated (active) β-catenin antibody in retinal sections from fish treated with DMSO (vehicle control) or 1-azakenpaullone from 24 to 48 hpf. Average intensity was calculated from approximately 750 μm^2^ area either in central retina or peripheral retina. DMSO-treated controls show significantly more active β-catenin in peripheral retina compared to central retina, while fish treated with 1-azakenpaullone have elevated levels of active β-catenin in central retina, comparable to that in control-treated peripheral retina. **(B-E)** Dominant-negative TCF3 suppresses the expansion of the CMZ induced by GSK inhibition. *Tg*(*hsp70:ΔTCF3-GFP*)^*w26*^ zebrafish embryos, which express a dominant-negative TCF3 (dnTCF) under control of the heat shock promoter, and their wild-type siblings were heat shocked at 36 hpf for 1 h at 39.5°C and then treated with either 0.025% DMSO or 2.5 μM 1-azakenpaullone. Fish were given a pulse of BrdU 2 h prior to fixation at 3 dpf. **(B)** Wild-type siblings treated with DMSO show a normal CMZ, with BrdU-positive and PCNA-positive cells at the retinal margin. **(C)** DMSO-treated fish expressing dnTCF showed a decrease in the number of cells at the retinal margin that were BrdU/PCNA immunoreactive. **(D)** In wild-type sibling fish treated with 1-azakenpaullone, there is a dramatic expansion of the proliferative progenitors marked with PCNA and BrdU. **(E)** Transgenic fish expressing dnTCF and treated with 2.5 μM 1-azakenpaullone show no expansion of the CMZ, with few dividing cells at the retinal margin, indicating that dnTCF suppresses the 1-azakenpaullone induced expansion of the progenitor pool. Scale bar = 100 μm.

**Figure 6:**
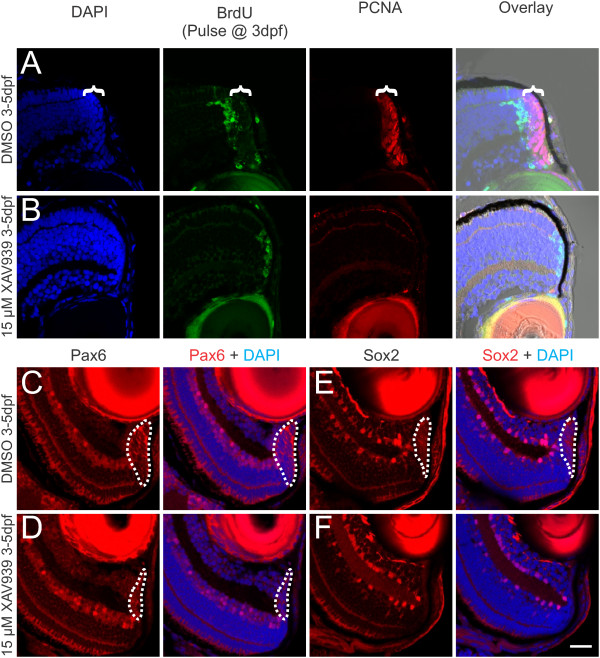
**Blocking Wnt signaling with XAV939 leads to loss of the progenitor population in the CMZ.** Zebrafish at 3 dpf were given a pulse of BrdU to mark dividing cells, then exposed to either 0.15% DMSO as a vehicle control or 15 μM XAV939 for 2 days and fixed for analysis at 5 dpf. In control fish at 5 dpf, the progeny of cells labeled with BrdU at 3 dpf had differentiated and were displaced out of the CMZ and a population of dividing cells that express PCNA remain in the CMZ (bracket) at 5 dpf **(A)**. In fish treated with 15 μM XAV939, the progeny of the cells labeled with BrdU at 3 dpf remain at the retinal margin and there are no PCNA-labeled cells **(B)**. Overlays in A and B are shown including a brightfield image to allow visualization of the RPE marking the edge of the retina. Labeling control **(C, E)** or XAV939-treated **(D, F)** fish with antibodies against Pax6 **(C, D)** or Sox2 **(E, F)**, shows that XAV939 greatly reduces the number of cells expressing these progenitor cell markers (dotted line marks the extent of labeling in the CMZ). Scale bar = 20 μm.

To confirm that the retinal effects of 1-azakenpaullone were due to its actions on the β-catenin/Wnt signaling cascade, rather than other pathways modulated by GSK3β, we first quantified the amount of dephosphorylated (active) β-catenin to test whether 1-azakenpaullone was resulting in a functional increase in active β-catenin. We labeled retinal sections from fish treated with either DMSO or 1-azakenpaullone from 24 to 48 hpf with an antibody against active β-catenin, with labeling and imaging done with identical parameters. When we compared the average fluorescence intensity in 1,000 μm^2^ regions from the central retina (containing cells beginning to differentiate by 48 hpf) or peripheral retina (comprising the CMZ and cells where the wave of differentiation has not yet reached), control retinas had more than twice as much labeling with the anti-active β-catenin antibody in the peripheral retina as they did in central retina (Figure 5A; *n* = 10 retinas; *P* <0.001; *T*-test), consistent with active Wnt-signaling at the periphery of the retina. Retinas from fish treated with 1-azakenpaullone had approximately the same level of active β-catenin in their central retina as controls did in their peripheral retina (Figure 6A; *n* = 10 retinas, *P* > 0.4; *T*-test). This is consistent with 1-azakenpaullone inhibiting GSK3β, preventing the phosphorylation and degradation of β-catenin, hyperactivating the canonical Wnt signaling pathway in regions of central retina that would have normally begun to repress β-catenin and differentiate.

To further confirm that this 1-azakenpaullone-mediated activation of Wnt/β-catenin signaling was responsible for maintaining retinal cells in a progenitor state, we used a genetic method to block Wnt signaling downstream of β-catenin with the transgenic line *Tg*(*hsp70:ΔTCF-GFP*)^*w26*^ - a heat-shock-inducible dominant-negative TCF3 (dnTCF), which lacks the β-catenin binding site. Activation of the Wnt signaling cascade leads to stabilization of β-catenin, which enters the nucleus, binds to TCF leading to a change in expression of Wnt target genes [28]; as the dnTCF lacks the β-catenin binding site, it should suppress the 1-azakenpaullone-mediated maintenance of proliferation if the primary effect of 1-azakenpaullone was via upregulation of active β-catenin. Transgenic embryos and wild-type siblings at 36 hpf were heat shocked for 1 h at 39.5°C to induce the transgene, then treated with either 0.025% DMSO or 2.5 μM 1-azakenpaullone until 72 hpf, when fish were incubated in BrdU to label dividing cells, and fixed after 2 h. Wild-type siblings treated with DMSO showed a normally proliferative CMZ, as marked by BrdU and PCNA immunoreactivity (Figure 5B). Wild-type siblings treated with 1-azakenpaullone showed the expanded zone of BrdU + cells passing through S-phase at 72 hpf (Figure 5D). In contrast, *Tg*(*hsp70:ΔTCF-GFP*)^*w26*^ embryos treated with DMSO after heat-shock had a smaller CMZ (Figure 5C), and those treated with 1-azakenpaullone also had few cells in the CMZ labeled with BrdU and PCNA (Figure 5E). We occasionally noted a few BrdU^+^ cells in the dorsal ONL of heat-shocked dnTCF expressing embryos (for example, Figure 5C). As Wnt-signaling is required for appropriate specification and maintenance of dorsal retinal identity, and dorsal marker expression can be lost with inhibition of Wnt signaling in the same time frame as these experiments [29], it is possible that the orderly wave of differentiation is slightly disrupted in these retinas. Most critically, though, inhibition of Wnt signaling via the dominant-negative TCF is able to suppress proliferation within the CMZ, and in particular is able to prevent the expansion of the CMZ induced by 1-azakenpaullone-mediated inhibition of GSK3β.

### Wnt signaling is required for ongoing retinal neurogenesis

Since stimulation of Wnt signaling leads to an expansion of the CMZ, these results suggest that Wnt signaling may function to maintain the progenitor population. To test this hypothesis, we treated zebrafish with the tankyrase inhibitor XAV939, which inhibits the transduction of a Wnt signal to GSK3β by stabilizing, axin a negative regulator of the Wnt signaling pathway [30]. We allowed embryos to develop to 3 dpf, to complete initial retinal differentiation, at which point the cells in the CMZ are the only proliferative cells (Figure 3D). Fish were given a 10-min pulse of 10 mM BrdU at 3 dpf to label the pool of actively dividing cells at that time, then treated with either 15 μM XAV939 or 0.15% DMSO as a vehicle control, allowed to grow for 2 days and fixed and processed for immunocytochemistry. In control zebrafish, the cells of the CMZ continue to proliferate, displacing post-mitotic cells out of the CMZ and into the retina. These post-mitotic cells retain BrdU, while actively dividing cells dilute out the BrdU label with additional rounds of S-phase. Thus in control retinas, cells labeled with BrdU at 3 dpf are just inside the CMZ at 5 dpf, adjacent to the actively dividing population in the CMZ that continue to express PCNA (Figure 6A). In contrast, in fish treated with 15 μM XAV939 to inhibit the transduction of Wnt signaling, the BrdU-positive cells remain at the peripheral margin of the retina and no cells are PCNA-positive, suggesting that in the absence of Wnt signaling there was very limited cell division following the BrdU pulse at 3 dpf, and most of the progeny of the cells labeled with BrdU at 3 dpf have exited the cell cycle (Figure 6B).

To test whether these cells also stopped expressing retinal progenitor cell markers in response to inhibition of Wnt signaling, we examined both Pax6 and Sox2 expression in the CMZ of control and XAV-treated fish, as Pax6 is highly expressed in the CMZ [2] and Sox2 is also expressed in the CMZ and has been shown to be regulated by Wnt signaling in *Xenopus*[11,16]. While a large pool of cells at the CMZ strongly express Pax6 in DMSO-treated control fish (Figure 6C), fish treated with XAV939 show a marked reduction in the size of this pool and in the level of Pax6 expressed (Figure 6D). We measured the area of the retinas containing Pax6-expressing cells in two sections from each of five control and XAV939-treated fish to quantify the change in the size of the CMZ. In DMSO-treated retinas, the Pax6-expressing area at the retinal margin was 717.1 ± 43.0 μm^2^ while in fish treated with XAV939 from 3 dpf to 5 dpf, the Pax6 expressing area was only 284.9 ± 23.5 μm^2^. Thus, XAV939 results in a significant decrease in the cross-sectional area of the CMZ (*P* < <0.001; *T*-test). Similarly, progenitor cells in the CMZ express Sox2 in DMSO-treated controls (Figure 6E), but there is little or no expression of sox2 in XAV939-treated zebrafish (Figure 6F). Thus, in the absence of Wnt signaling, there is both a loss of proliferative cells in the CMZ and a loss of expression of retinal progenitor markers, suggesting that Wnt signaling is necessary to maintain the pool of retinal progenitors at the retinal margin.

### Activation of Wnt signaling is necessary for photoreceptor regeneration

**Figure 7:**
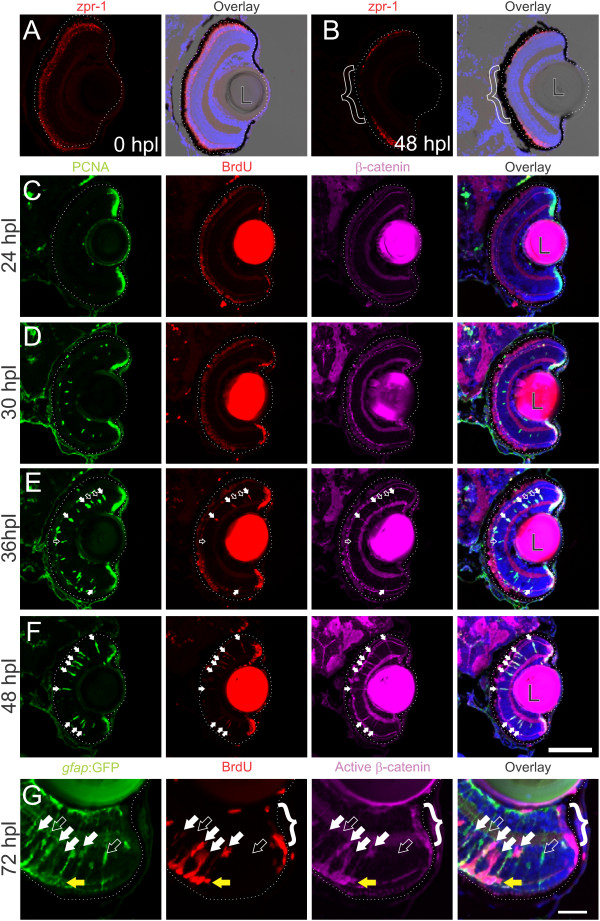
**Wnt signaling is activated as Müller glia reenter the cell cycle following intense-light-induced destruction of photoreceptors.***Tg*(*gfap:GFP*)^*mi2002*^ zebrafish larvae were exposed to intense light at 6 dpf to lesion their photoreceptors. Immediately following exposure, the retina appeared normal, with photoreceptors (double cones labeled with zpr-1) throughout the ONL **(A)**. Within 48 hours post lesion (hpl), photoreceptors are missing from central retina as shown by the lack of zpr-1 staining (**B**; bracket). **(C-F)** Light-lesioned fish were incubated continuously in 2.5 mM BrdU and fixed at 24, 30, 36, or 48 hpl. Cells in the INL become PCNA-positive between 24 and 30 hpl, and begin to incorporate BrdU around 36 hpl **(C-E)**. Immunoreactivity for β-catenin is absent from the INL at 24 or 30 hpl **(C, D)**, but begins to accumulate by 36 hpl **(E)**, when all BrdU-positive, PCNA-positive cells (white arrows) and some BrdU-negative, PCNA-positive cells (open arrows) are immunoreactive. By 48 hpl, all of the PCNA-positive cells are also BrdU-positive and β-catenin-positive (**F**; white arrows). At 72 hpl, BrdU-positive cells in the INL also express GFP, indicating their Müller glial origins, and they are co-labeled with an antibody for dephosphorylated (active) β-catenin (white arrows; **G**). Proliferative cells are strongly immunoreactive for dephosphorylated β-catenin (open arrow). The cells of the CMZ also exhibit strong immunoreactivity for dephosphorylated β-catenin (bracket). Scale bars **A-F**: 100 μm; G: 50 μm.

Given that Wnt signaling is necessary for maintaining retinal progenitor cells in the CMZ in a proliferative state, and has been implicated in proliferation and regeneration of retinal neurons following stab lesions to the retina [19], we next asked whether Wnt signaling was also reactivated during the regenerative response to selective loss of photoreceptors in larval fish. Intense light from a metal-halide lamp for 20 min induces a rapid, photoreceptor-selective lesion in the central retina of 6-day-old zebrafish larvae (Figure 7A, B). Differentiated Müller glia, which have nuclei in the INL and radial processes that span the width of the retinal epithelium, respond locally to retinal injury by dedifferentiation and reentry into the cell cycle. The nuclei of these activated Müller glia migrate apically into the ONL, where they undergo an asymmetric, self-renewing division in which one daughter becomes a neuronal progenitor that lacks apical/basal processes and proliferates rapidly to produce a cluster of progenitors surrounding the Müller glial cell; these Müller-derived progenitors will in turn regenerate photoreceptors [4,5,31]. Consistent with this proliferative response, we observed expression of PCNA in nuclei in the INL and ONL beginning between 24 and 30 h post lesion (hpl) (Figure 7C, D). These cells began to accumulate BrdU around 36 hpl, indicating that they had progressed into S-phase (Figure 7E), and by 48 hpl PCNA-positive and BrdU-positive neurogenic clusters spanned the INL and ONL (Figure 7F).

We next assayed activation of Wnt signaling in the injury-induced Müller glia by looking for stabilization of β-catenin; when Wnt signaling suppresses GSK3β activity, β-catenin becomes dephosphorylated, blocking its ubiquitination and destruction, allowing it to accumulate in the cytoplasm and nucleus. We did not observe β-catenin immunoreactivity in retinal cells at 24 or 30 hpl, but it began to accumulate in the proliferative cells by 36 hpl (Figure 7E). β-catenin accumulated in all BrdU-positive cells at 36 hpl (filled arrows in Figure 7E), and was also found in occasional PCNA-positive, BrdU-negative cells (open arrows in Figure 7E). By 48 hpl, all PCNA positive cells within the region of the lesion were BrdU-positive and had strong labeling of cytoplasmic β-catenin (filled arrows in Figure 7F). To confirm that this accumulation of β-catenin was due to a lack of phosphorylation by GSK3β, we used an antibody selective for dephosphorylated, active β-catenin [32]. We again found selective immunolabeling for dephosphorylated β-catenin in the proliferating Müller glia (marked by expression of the *gfap*:GFP transgene and incorporation of BrdU; white arrows in Figure 7G) but not in non-proliferative Müller glia (GFP-positive, BrdU-negative; open arrows in Figure 7G) at 72 hpl. We also observed immunolabeling of dephosphorylated β-catenin in the proliferative cells in the ONL, which also had residual *gfap*:GFP transgene expression indicating that they were the progeny of the proliferative Müller glia (yellow arrow; Figure 7G). Additionally, dephosphorylated β-catenin accumulated in the cells of the CMZ (bracket; Figure 7G), consistent with ongoing Wnt-signaling at the retinal margin.

**Figure 8:**
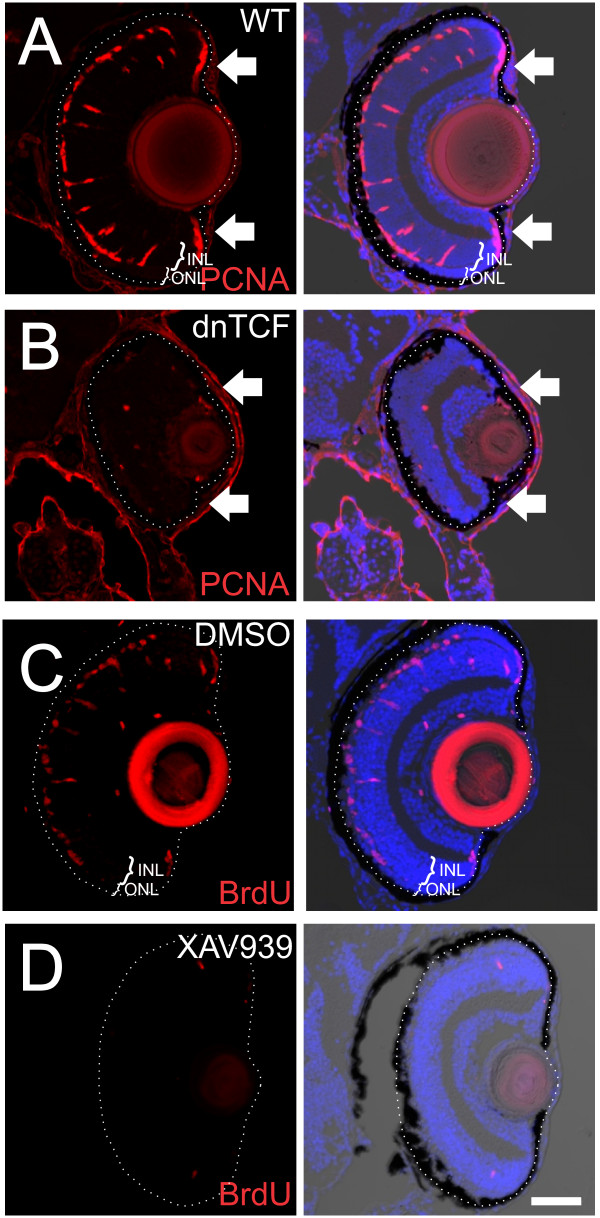
**Inhibition of Wnt signaling with dnTCF or XAV939 inhibits injury-induced proliferation. (A, B)** dnTCF zebrafish larvae and wild-type siblings were given an intense light lesion at 6 dpf and heat shocked at 39.5°C for 1 h at 1 dpl and again at 2 dpl then fixed at 3 dpl. In wild-type siblings, PCNA-labeled proliferative cells are in the CMZ (arrows), INL, and ONL at 3 dpl **(A)**, while dnTCF zebrafish have few proliferative cells in either the retina or the CMZ **(B)**. **(C, D)** Wild-type zebrafish lesioned as in **(A, B)** were treated with 0.15% DMSO or 15 μM XAV939, with 2.5 mM BrdU present continuously in the media until fixation at 3 dpl. In DMSO-treated fish, BrdU-positive cells are in the INL and ONL **(C)**, while XAV939-treated fish have few proliferative cells in either the INL or ONL **(D)**. Scale bar = 50 μm.

In order to test whether activation of Wnt signaling was required for endogenous and/or injury-induced proliferation in the retina, we light-lesioned *Tg*(*hsp70:ΔTCF-GFP*)^*w26*^ larval zebrafish and wild-type siblings, and heat-shocked the fish for 1 h at 1 dpl and again at 2 dpl to induce the dominant negative TCF; fish were fixed at 3 dpl. Whereas wild-type siblings had normal expression of PCNA in the INL and ONL in the region of the lesion at 3 dpl (Figure 8A), transgenic fish expressing dnTCF had few to no PCNA-positive cells in either the INL or ONL (Figure 8B). Notably, these fish also had few, if any, proliferative cells in the CMZ compared with controls (arrows; Figure 8A, B). We quantified the number of PCNA-positive cells within the lesioned retina at 3 dpl, normalizing it to the linear length of the lesion to reduce variation caused by differences in the extent of photoreceptor loss. Wild-type siblings had 11.9 ± 0.6 PCNA-positive cells per 100 μm of lesioned retina while dnTCF fish had 1.4 ± 0.2 PCNA-positive cells per 100 μm of lesioned retina (*n* = 6 fish; mean ± s.e.m.), which was a statistically significant difference (*t*-test; *P* < <0.0001). Similarly, pharmacological block of Wnt signaling by treatment of light-lesioned fish with 15 μM of the tankyrase inhibitor/axin stabilizing agent XAV939 from 0 to 3 dpl resulted in few to no proliferating cells (including in the CMZ), compared with DMSO-treated controls (Figure 8C, D). Thus, either pharmacological or genetic inhibition of Wnt signaling inhibits the injury-induced activation of Müller glial proliferation.

### Activation of Wnt signaling during photoreceptor regeneration leads to loss of Müller glia

**Figure 9:**
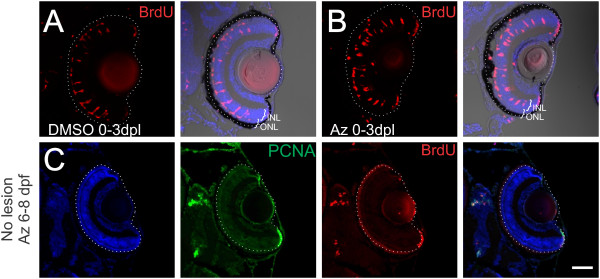
**Inhibition of GSK3β does not alter number of dedifferentiating Müller glia.** Zebrafish larvae were exposed to intense-light at 6 dpf and incubated in BrdU with either DMSO as a vehicle control or 1-azakenpaullone from 0 to 3 dpl. **(A, B)** Light-lesioned fish treated with DMSO or 1-azakenpaullone from 0 to 3 dpl had no difference in the number of BrdU-positive cells in the INL and in the ONL. **(C)** Zebrafish that did not have a light lesion were soaked in 2.5 μM 1-azakenpaullone from 6 to 8 dpf to test whether it was sufficient to induce Müller glial proliferation in the absence of a lesion. We saw no PCNA or BrdU-positive nuclei in unlesioned fish. Scale Bar = 100 μm.

We next asked whether constitutive activation of Wnt signaling using 1-azakenpaullone would alter the regenerative response in Müller glia induced by selective loss of photoreceptors. In light-lesioned *Tg*(*gfap:GFP*)^*mi2002*^ fish treated with 2.5 μM 1-azakenpaullone from 0 to 3 dpl cell proliferation in the INL and ONL was comparable to controls (Figure 9A, B). The number of BrdU-positive cells in the INL (indicative of Müller glia reentering the cell cycle) in control versus 1-azakenpuallone-treated retinas was not significantly different (5.0 ± 0.5 BrdU-positive cells in the INL per 100 μm of lesioned retina for control; 4.8 ± 0.6 for 1-azakenpaullone-treated; *P* > 0.5). A previous study in adult zebrafish retina showed that intraocular injection of a GSK3β inhibitor was sufficient to stimulate dedifferentiation and proliferation of Müller glia in an uninjured retina [19], so we next asked whether soaking larval fish at 6 dpf in 1-azakenpaullone and BrdU for 48 h was sufficient to induce a similar response. However, we did not see any proliferating cells (either BrdU-positive or PCNA-positive) within the central retina of the treated larval zebrafish (Figure 9C), suggesting that activation of Wnt/β-catenin signaling is not sufficient to induce Müller glial dedifferentiation and proliferation in the absence of injury in larval retinas.

**Figure 10:**
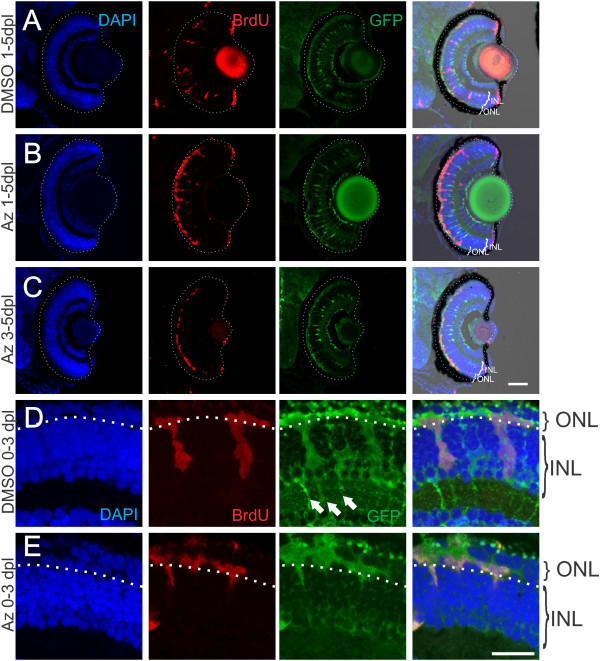
**Inhibition of GSK3β results in delayed loss of proliferative Müller glia.***Tg*(*gfap:GFP*)^*mi2002*^ zebrafish larvae were exposed to intense-light at 6 dpf and incubated in BrdU with either DMSO as a vehicle control or 1-azakenpaullone from 1 to 5 dpl or 3 to 5 dpl. **(A)** Light-lesioned fish treated with DMSO from 1 to 5 dpl have BrdU-positive cells in the INL and in the ONL, and a normal distribution of GFP-labeled Müller glia in the INL. **(B)** In light-lesioned fish treated with 1-azakenpaullone from 1 to 5 dpl, BrdU-positive cells are only in the ONL, with few-to-none in the INL. Additionally, fewer GFP-labeled Müller glia are present compared to controls (see results). **(C)** Light-lesioned zebrafish treated with BrdU from 1 to 5 dpl and 1-azakenpaullone from 3 to 5 dpl also have few or no BrdU-positive cells in the INL and the number of Müller glia is reduced. **(D, E)** High magnification views of BrdU-positive GFP-positive cells that accumulated in the ONL at 3 dpl. **(D)** In fish treated with DMSO from 0 to 3 dpl, clusters of GFP-positive, BrdU-positive Müller glia and their progeny are distributed between both the ONL and the INL (dotted line indicates the outer plexiform layer separating the ONL and the INL). Arrows indicate the GFP-labeled basal processes of radial Müller glia. **(E)** In fish treated with 2.5 μM 1-azakenpaullone from 0 to 3 dpl, most of the BrdU-positive GFP-positive cells are in the ONL, and the radial processes of Müller glia are absent. Scale bar: A-C, 100 μm; D, E, 20 μm.

Surprisingly, when light-lesioned fish were treated with azakenpaullone and BrdU from 1 to 5 dpl, we found a notable difference in the pattern of BrdU-positive nuclei. BrdU was continuously available in the media from 1 to 5 dpl, to mark all cells that divided and their progeny. Control fish had BrdU-positive nuclei in both the ONL and INL (Figure 10A). The BrdU-positive cells within the INL and ONL were GFP-positive when this experiment was done with *Tg*(*gfap:GFP*)^*mi2002*^ fish, indicating that the BrdU-positive cells were Müller glia or their neurogenic progeny. However, in fish treated with 1-azakenpaullone from 1 to 5 dpl with BrdU present throughout, many BrdU-positive cells were found in the ONL, but few BrdU-positive cells were in the INL compared with controls (Figure 10B). While control fish had 8.6 ± 0.8 BrdU-positive cells in the INL per 100 μm of lesioned retina, fish treated with 1-azakenpaullone had only 2.5 ± 0.6 BrdU-positive cells per 100 μm of lesioned retina (*n* = 5 fish each; mean ± s.e.m.). Thus 1-azakenpaullone treatment produced a significant decrease in the number of BrdU-positive cells in the INL (*t*-test; *P* < <0.001). In contrast, there was no difference between DMSO and 1-azakenpaullone treated fish in the number of BrdU-positive cells in the ONL at 5 dpl (10.6 ± 0.9 cells per 100 μm and 9.4 ± 1.7 cells per 100 μm, respectively; *P* > 0.5).

Since treatment with 1-azakenpaullone from 0 to 3 dpl resulted in numerous BrdU-positive cells within the INL and ONL, and since the nuclei of activated Müller glia migrate apically to initiate mitosis in the ONL, these data suggest that the decrease in BrdU-positive cells in the INL after 5 days of treatment with 1-azakenpaullone is not due to failure of the Müller glia to initially re-enter the cell cycle, but instead affects a later stage in regeneration. To confirm this interpretation, we treated light-lesioned fish with BrdU from 1 to 5 dpl to label all dividing cells, but exposed them to 1-azakenpaullone only from 3 to 5 dpl. These fish again had few BrdU-positive cells within the INL, although BrdU-positive cells were plentiful within the ONL (Figure 10C). These data, together with the finding of BrdU-positive cells in the INL in fish treated with 1-azakenpaullone from 0 to 3 dpl, suggest that 1-azakenpaullone does not prevent the initial activation of Müller glia and their re-entry into the cell cycle.

Since we observed fewer BrdU-positive cells within the INL at 5 dpl in light-lesioned, 1-azakenpaullone-treated fish, and since the nuclei of Müller glia return to the INL (after their initial apical migration into the ONL), we next asked whether the lack of BrdU-labeled progeny in the INL indicated a loss of Müller glia from the INL. At 5 dpl DMSO-treated fish had 8.8 ± 0.5 GFP-positive Müller glia within the lesioned area of the INL per 100 μm of lesion, and these cells exhibited regular spacing (Figure 10A). The fish treated with 1-azakenpaullone from 1 to 5 dpl had significantly fewer, irregularly-spaced, GFP-positive Müller glia within the lesioned area of the INL (Figure 10B; 5.8 ± 0.5 cells per 100 μm of lesion; *P* < 0.005 by *t*-test).

These observations suggest that when β-catenin/Wnt signaling is constitutively activated in light-lesioned retinas, Müller glia are either being removed from the retina by cell death, or that the Müller glia cell divisions are no longer asymmetric or self-renewing (that is, producing a Müller glial cell plus a neuronal progenitor) but instead both daughter cells are diverted into the retinal progenitor fate and remain in or move into the ONL. TUNEL-labeling on 1-azakenpaullone-treated fish at 3 dpf to mark cells undergoing apoptosis, revealed no TUNEL-labeled cells within the INL, suggesting that 1-azakenpaullone does not stimulate significant cell death of dividing Müller glia (data not shown).

To examine whether 1-azakenpaullone leads to a change in progenitor fate, we examined the Müller glial-derived clusters of proliferative cells at 3 dpl. In control fish, the GFP-positive, BrdU-labeled cell clusters span the ONL and INL and the radial process of Müller glia that extend basally from the INL into the inner retina are clearly visualized (Figure 10D). In contrast, in 1-azakenpaullone-treated fish, the GFP-positive, BrdU-labeled cells are largely confined to the ONL, and basal processes of Müller glia are absent (Figure 10E). This suggests that activation of Wnt-signaling blocks the asymmetric, self-renewal division of injury-induced Müller glial cells, and instead biases all the Müller-glial derived progeny to move into the ONL where they may act as retinal progenitors.

Together, these data demonstrate that β-catenin/Wnt signaling is initially required to activate cell-cycle re-entry in Müller glia following injury and that Wnt signaling subsequently controls the fate of the progeny of those cell divisions, biasing them to produce retinal progenitors at the expense of self-renewal to retain the original Müller glial population.

## Discussion

We have used genetic and pharmacological manipulation of the canonical Wnt signaling cascade to show that Wnt signaling plays a key role in maintaining retinal progenitors in a proliferative state during both retinal growth and lesion-induced regenerative proliferation. Inhibition of canonical Wnt-signaling led to loss of retinal stem and progenitor cells in the CMZ and prevented cell cycle re-entry in Müller glia following photoreceptor loss. Stimulation of the Wnt cascade by inhibition of GSK3β led to expansion of the CMZ during development and altered the fate decisions of Müller glial cells induced to proliferate following photoreceptor loss.

We found that activation of Wnt signaling by inhibition of GSK3β with 1-azakenpaullone, which has been previously used predominantly in cell culture, works well in zebrafish *in vivo* and phenocopies the effects on eye development seen in *axin1*-deficient fish [20,21]. 1-azakenpaullone is highly selective for GSK3β, though it can inhibit cyclin-dependent kinases (CDKs) at higher concentrations [25]. As the phenotype that we see is an increase in proliferation rather than an decrease that would be expected if 1-azakenpaullone was inhibiting CDKs, and since the effects of 1-azakenpaullone can be blocked with dominant-negative TCF which is downstream of GSK3β in the Wnt signaling cascade, we conclude that the primary effect of 1-azakenpaullone in our system is inhibition of GSK3β to stimulate the β-catenin/Wnt cascade. We have also used XAV939, a tankyrase inhibitor that stabilizes axin [30], and showed that it phenocopies the effects of a dominant-negative TCF3 transgene. These pharmacological agents therefore allow the activation or inhibition of the β-catenin/Wnt cascade at controlled dosages and precise timings allowing us to use consistent methods to examine the role of Wnt signaling across initial retinal development, ongoing growth from the CMZ and regeneration following photoreceptor damage.

### β-catenin/Wnt signaling is sufficient and necessary to maintain retinal progenitor fate in the CMZ

The canonical Wnt signaling pathway is well-established as a key mediator of stem cell identity in tissues with ongoing growth. In many epithelial stem cells, including those in intestinal crypts, mammary glands, and skin, Wnt signaling is active in the stem cell niche and is necessary for maintaining stem cell identity [7,8,33]. Hyperactivation of Wnt signaling leads to expansion of the stem cell pool and blocks differentiation of the stem cell progeny, and such hyperactivation is found in many cancers [8,33]. Inhibition of Wnt signaling by blocking the interaction of β-catenin with TCF results in loss of the stem cell pool [33]. Our data fit this general model, and paired with several other studies, suggest that β-catenin/Wnt signaling also maintains a proliferative progenitor cell pool within the CMZ in the retina of fish, amphibians, and chicks [9,11,12,14-16]. While hyperactivation of β-catenin/Wnt signaling before or during gastrulation inhibited forebrain and eye formation due to the early posteriorizing effects of Wnt [34], stimulation of β-catenin/Wnt signaling after gastrulation inhibited differentiation of the retina and maintained the cells in a proliferative progenitor state. The pharmacologically induced expansion in the expression domain of retinal stem/progenitor cell markers is similar to what has been reported in fish with a mutation in the adenomatous polyposis coli (APC) protein, which leads to overactivation of the Wnt/β-catenin pathway [14]. We also found that dephosphorylated β-catenin accumulates in the cytoplasm of progenitor cells in the CMZ, confirming this as a zone of ongoing, canonical Wnt signaling [32]. Inhibition of Wnt signaling, via either dominant-negative TCF or XAV939, leads to loss of proliferative cells at the retinal margin and in loss of expression of retinal progenitor markers at the CMZ, consistent with data from *Xenopu*s suggesting that Wnt signaling maintains retinal progenitors by regulating sox2 expression [11,16]. Thus we demonstrate that β-catenin/Wnt signaling is sufficient and necessary to maintain retinal progenitor identity in zebrafish.

### β-catenin/Wnt signaling is necessary for retinal regeneration

β-catenin/Wnt signaling is also implicated as a key regulator of the stem cell state in many regenerating systems, such as zebrafish and amphibian tail [35,36], where activating β-catenin/Wnt signaling promotes proliferation of stem cells and inhibition prevents regeneration. Although the mammalian retina does not exhibit a robust regenerative response, activation of Wnt signaling stimulates Müller glial cells to reenter the cell cycle in retinal explants *in vitro*[17,18]. However, it is not clear from those studies whether Wnt signaling primarily affects Müller glial proliferation or dedifferentiation or whether Wnt signaling is required for Müller glial-dependent regeneration. Recent work by Ramachandran *et al.*[19] reported that β-catenin accumulated in dedifferentiating Müller glia following stab lesions to the retina, and that activation of Wnt signaling was necessary for the proliferation of these Müller glia. We, therefore, tested whether Wnt signaling is also reactivated in response to the selective loss of photoreceptors in the zebrafish retina, where there is a rapid and robust dedifferentiation and proliferation of Müller glia following injury [4]. We have modified the intense light-lesioning protocol that we previously used in adult zebrafish [4,37] to allow for the rapid (20 min) destruction of photoreceptors in larval zebrafish as early as 6 dpf. The lesion results in a reproducible and rapid loss of photoreceptors from a large extent of central retina. As this method allows the rapid lesioning of many larvae simultaneously, it has significant advantages for future studies utilizing high-throughput screening studies; for example, to identify small molecules that are neuroprotective or promote regeneration.

Müller glia re-enter the cell cycle as early as 24 hpl following intense light damage of retinal photoreceptors, but cytoplasmic expression of β-catenin is not apparent until 36 hpl. The delay between cell cycle re-entry, as marked by PCNA, and β-catenin accumulation suggests either that Wnt signaling is activated secondary to the initial injury signal that induces dedifferentiation and proliferation of Müller glia, or that the levels of β-catenin at the onset of Müller glia proliferation are too low for immunocytochemical detection. In proliferating cells, PCNA is first expressed in G1 phase, preceding S-phase entry and BrdU accumulation [38], whereas Wnt regulates passage through the G1/S checkpoint via transcriptional activation of cyclin D1 [39], consistent with PCNA immunoreactivity preceding β-catenin accumulation. Notably, however, when we inhibited Wnt signaling by inducing expression of dominant-negative TCF or with the tankyrase inhibitor XAV939, we observed few PCNA-positive or BrdU-positive cells following light damage, consistent with what was reported for Müller glial proliferation following stab lesions [19]. Thus, Wnt signaling is either necessary for the initial expression of PCNA as the Müller glia return to the cell cycle, or, alternatively, when Wnt signaling is blocked, activated Müller glia do not accumulate PCNA as they cannot proceed through the cell cycle. These data show that β-catenin/Wnt signaling is required for injury-induced Müller glial proliferation and generation of retinal progenitors. Consistent with this interpretation, dephosphorylated β-catenin is maintained in the proliferative progenitor cells in the ONL.

Our data suggest that stimulation of Wnt signaling by soaking larval zebrafish for 2 days in the GSK3β inhibitor 1-azakenpaullone (from 6 to 8 dpf) is not sufficient to trigger dedifferentiation and proliferation of Müller glia, in contrast to what was reported for intraocular injection of a different GSK3β inhibitor into the eyes of adult zebrafish [19]. Additionally, in our experiments applying 1-azakenpaullone during development, we find that 1-azakenpaullone can maintain already proliferating progenitors in an undifferentiated state, but does not lead differentiated Müller glia to re-enter the cell cycle (for example, Figure 3, Additional file 1: Figure S1). In our lesion experiments with 1-azakenpaullone, we only see proliferative cells within and adjacent to the lesioned area, and do not see them in unlesioned regions (for example, the BrdU-labeling in Figure 10C extends only within the region where the photoreceptor nuclei are lost; where the ONL appears normal in the ventral retina, there are no more BrdU^+^ cells in the INL or ONL). The difference between our data and the previous report may be due to the method for administering the inhibitor: intraocular injection used in the previous report [19] is an invasive procedure that inevitably produces trauma in the eye (and perhaps the retina) and may have provoked an injury response in the Müller glia, in contrast to the method used in the present study, soaking the larval fish in the drug. Alternatively, the difference may result from the age of the fish. While further experimentation will need to be done to clarify the role of Wnt signaling on Müller glia, our data suggest that Wnt signaling may not be sufficient to trigger dedifferentiation and a return to the cell cycle in uninjured zebrafish retinas.

In zebrafish, where injury-induced Müller glial proliferation is rapid and robust in the retinas of both larval and adult fish, our data show that constitutive activation of Wnt signaling does not enhance the initial proliferative response of Müller glia following damage. However, prolonged activation of β-catenin/Wnt signaling alters the fate of the Müller-glia-derived progenitors. The initial mitotic event is an asymmetric, self-renewing division: one daughter retains the identity as a Müller glial cell and the other daughter continues to proliferate, generating a cluster of neuronal progenitors that remain associated with the radial process of the originating Müller glia [4,5,37]. In contrast, after treatment with GSK3β inhibitor, all of the progeny of mitotic divisions of Müller glia accumulate in the ONL depleting the population of differentiated Müller glia. This suggests that activation of Wnt signaling may lead to a symmetric division in which both cells take on a progenitor fate and move into the ONL. At present there are few markers that distinguish between progeny of Müller glia that will return to being Müller glia or that will be progenitors in the ONL. As additional work clarifies the normal gene expression and fate of these progeny, it will be important to test how Wnt signaling alters those fates. Importantly, recent work on the role of Wnt signaling in neurogenesis and radial glia in post-embryonic zebrafish hypothalamus found results very similar to ours; in particular, stimulation of Wnt signaling increased the number of neurogenic progenitors, but also resulted in loss of radial glial cells [40]. As the radial glia are thought to be the source for neurogenic progenitors in the zebrafish brain, including the hypothalamus [40-42], this is consistent with a broader model in which active Wnt signaling promotes a neural or retinal progenitor fate at the expense of a radial glial fate.

Although we have been unable to maintain fish in 1-azakenpaullone for longer than 5 days after lesion, likely due to the systemic effects of GSK3β-inhibition and hyperactivation of Wnt signaling, it will be important to explore the longer-term fates of these Wnt-activated progenitors as techniques allow more refined regulation of Wnt signaling, such as selective activation only in the Müller glia and their progeny. In particular, it will be important to determine whether these progenitors continue cycling, arrest, or retain the capacity to differentiate into replacement photoreceptors if Wnt signaling is subsequently suppressed.

## Conclusion

In this study, we provide evidence that, in zebrafish, β-catenin/Wnt signaling acts on two distinct populations of retinal stem/progenitor cells: (1) within the CMZ associated with normal retinal growth; and (2) activated Müller glia in the regenerating retina that are induced to divide and generate neuronal progenitors following ablation of photoreceptors with intense light. These data support the hypothesis that the CMZ progenitor cells and the injury-induced Müller glial cells and their progeny share many of the same regulatory networks [2]. β-catenin/Wnt signaling has also been found at the retinal margin of the mammalian retina [43], where the cells are not normally proliferative but can be induced to function like retinal stem cells in cell culture [44], and stimulation of the canonical Wnt pathway appears to increase proliferation of postnatal mammalian Müller glia [17,18]. Therefore, a better understanding of how Wnt signaling directs the decision of retinal progenitors whether to divide or to differentiate in robustly regenerating systems such as the zebrafish may be important for future clinical applications to enhance endogenous regenerative mechanisms in damaged or diseased human retina. Our data, together with previous studies that similarly implicate β-catenin/Wnt signaling in the regulation of progenitor fate in other developing and regenerating systems, suggest that this pathway is highly conserved as a mechanism to maintain a population of adult stem cells for ongoing growth of epithelia and for regeneration and repair following injury.

## Methods

### Fish lines and general care

Wild-type fish were of the TL strain, and were obtained from the Zebrafish International Resource Center (ZIRC; Eugene, OR, USA). In *Tg*(*gfap:GFP*)^*mi2002*^ fish, GFP is expressed in astroglial cells, including retinal Müller glia [26]. *Tg*(*hsp70:ΔTCF-GFP*)^*w26*^ fish have a heat-shock-inducible dominant negative TCF3 construct tagged with GFP [28], and were obtained from Richard Dorsky. *masterblind* (*mbl*) fish have a loss-of-function mutation in *axin1*[21], obtained from ZIRC. Fish were maintained on a 14:10 light:dark cycle at 28.5°C unless otherwise stated. All care and use of fish was approved by the Animal Care and Use Committees of the University of Michigan and Colgate University.

### Pharmacological manipulation of Wnt signaling

To stimulate Wnt signaling, zebrafish embryos were incubated in varying concentrations of the glycogen synthase kinase 3b inhibitor 1-azakenpaullone (Calbiochem; 10 mM stock in DMSO) diluted in E2 medium (15 mM NaCl, 0.5 mM KCl, 1 mM MgSO_4_, 0.15 mM KH_2_PO_4_, 0.05 mM Na_2_HPO_4_, 1 mM CaCl_2_, 0.7 mM NaHCO_3_; [45]); control embryos were treated with 0.1% DMSO. An alternative treatment for stimulation of Wnt signaling was incubation in E2 medium containing 0.3 M LiCl (Sigma-Aldrich) for 60 min followed by washout and culture in E2 medium. Wnt signaling was blocked with the tankyrase inhibitor/axin stabilizing agent XAV939 (Cayman Chemical; 10 mM stock in DMSO; [30]) diluted in E2. Dividing cells in embryos were labeled by incubation in ice-cold 5 mM BrdU (MPBIO, Solon, OH, USA) in 10% DMSO and 5 mM HEPES buffered E2 medium for 10 min; after washing three times in E2 media, embryos were returned to the incubator for 1 to 4 h prior to fixation. To label dividing cells in larvae after 5 dpf, fish were maintained continuously in 2.5 mM BrdU in 5 mM HEPES-buffered E2 medium, replaced every 2 days.

### Heat-shock induction of dnTCF

To induce dominant-negative TCF expression under control of the heat shock promoter in the Tg(hsp70:ΔTCF-GFP)^W26^ line, fish were heat shocked for 45 min to 2 h at 39.5°C.

#### Retinal lesions

High-intensity lesions of photoreceptors in larval zebrafish were done at 6 dpf. Fish were placed in a 50 mL beaker with 10 mL of E3 medium. A liquid fiber optic light line connected to a metal-halide microscope illuminator (X-Cite, Lumen Dynamics, Mississauga, ON, Canada; or EL6000, Leica Microsystems, Buffalo Grove, IL, USA) was positioned 2 cm from the edge of the beaker (4 cm from the center) such that the tip was in the center of the 10 mL water column. Light intensity was measured at approximately 1.5 x 10^5^ lux at the center of the beaker. Fish were illuminated for 20 min, then returned to culture as appropriate.

#### *Immunocytochemistry and* in situ *hybridization*

After fixation with 4% paraformaldehyde, zebrafish were cryoprotected in 20% sucrose in 0.1 M phosphate buffer, frozen in a 2:1 mixture of 20% sucrose in 0.1 M phosphate buffer:OCT (Tissue-Tek), and cryosectioned at 6 μm [46]. Exposure of PCNA, BrdU, and β-catenin antigens required antigen retrieval, and was done by boiling slides in 10 mM sodium citrate (pH 6.0) + 0.05% Tween-20 for 20 min, then allowing slides to cool to room temperature for 20 min before proceeding with standard immunocytochemistry. Following 1-h block with 10% normal goat serum in PBS with 0.1% Tween-20 (PBST), primary antibodies diluted in PBST were applied for 1 h at room temperature or overnight at 4°C. Primary antibodies and their dilutions included: rat anti-BrdU (Accurate), 1:50 dilution; mouse anti-BrdU BD, 1:50; mouse zpr-1 (Zebrafish International Resource Center), 1:200 dilution; mouse zpr-3 (Zebrafish International Resource Center), 1:200 dilution; mouse anti-PCNA (Sigma-Aldrich), 1:1,000 dilution; rabbit anti-GFP (Invitrogen), 1:400 dilution; mouse zrf-1 (Zebrafish International Resource Center), 1:200 dilution; rabbit anti-β-catenin (Anaspec), 1:100 dilution; mouse anti-active-β-catenin (Millipore), 1:250 dilution; rabbit anti-Pax6 (Anaspec); rabbit anti-Sox2 (GeneTex). *In situ* hybridization was performed as in Bernardos *et al.* (2007) on whole-mount embryos fixed in 4% PFA and stored in MeOH at −20°C prior to labeling with digoxigenin-tagged riboprobes for *rx1*[47], *vsx2*[48], and *sox2* (cb236 [49]).

## Abbreviations

APC: Adenomatous polyposis coli; BrdU: Bromodeoxyuridine; CDK: Cyclin-dependent kinase; CMZ: Ciliary marginal zone; dnTCF: Dominant negative T-Cell factor 3; GCL: Ganglion cell layer; GFAP: Glial fibrillary acidic protein; GFP: Green fluorescent protein; GSK3β: Glycogen synthase kinase 3β; INL: Inner nuclear layer; ONL: Outer nuclear layer; PCNA: Proliferating cell nuclear antigen.

## Competing interests

The authors declare that they have no competing interests.

## Authors’ contributions

JRM conceived and designed the study, conducted experiments, and wrote the manuscript. LH, AM, KK, and AP conducted experiments. PAR supervised the project and edited the manuscript. All authors read and approved the final manuscript.

## Supplementary Material

Additional file 1 **Figure S1.** Further evidence that inhibition of GSK3β blocks retinal differentiation and maintains proliferative progenitors. A pulse of BrdU provided 2 h prior to fixation at 72 hpf of fish treated with DMSO starting at 12 hpf shows the progenitors at the CMZ are actively moving through S-phase, while at the back of the retina zpr3 labels the rod photoreceptors **(A)**. Treatment with 2.5 μM 1-azakenpaullone beginning at 12 hpf prevents rod differentiation and BrdU-positive cells are found throughout the retina **(B)**. Treatment with 1-azakenpaullone beginning at 24 or 36 hpf allows some rods to differentiate, though there are still expanded pools of proliferating progenitors **(C, D)**. Fish treated with 1-azakenpaullone beginning at 24 hpf also have expanded PCNA-positive domains, loss of calretinin-positive neurons, loss of GFP-positive Müller glia, and loss of zpr-1 labeled double cones **(E, F)**. Inhibition of GSK3β with a 1 h treatment of 0.3 M LiCl at 48 hpf shows effects on retinal development similar to treatment with 1-azakenpaullone, with a reduced number of calretinin-positive neurons and an expanded CMZ (PCNA-labeled) compared with controls treated with 0.3 M NaCl **(G,H)**. Scale bar. Click here for file
