# Supplementary figures and images for: β-catenin/Wnt signaling controls progenitor fate in the developing and regenerating zebrafish retina
Source: Neural Dev. 2012 Aug 24;7:30. doi: 10.1186/1749-8104-7-30 (PMC3549768; doi:10.1186/1749-8104-7-30)

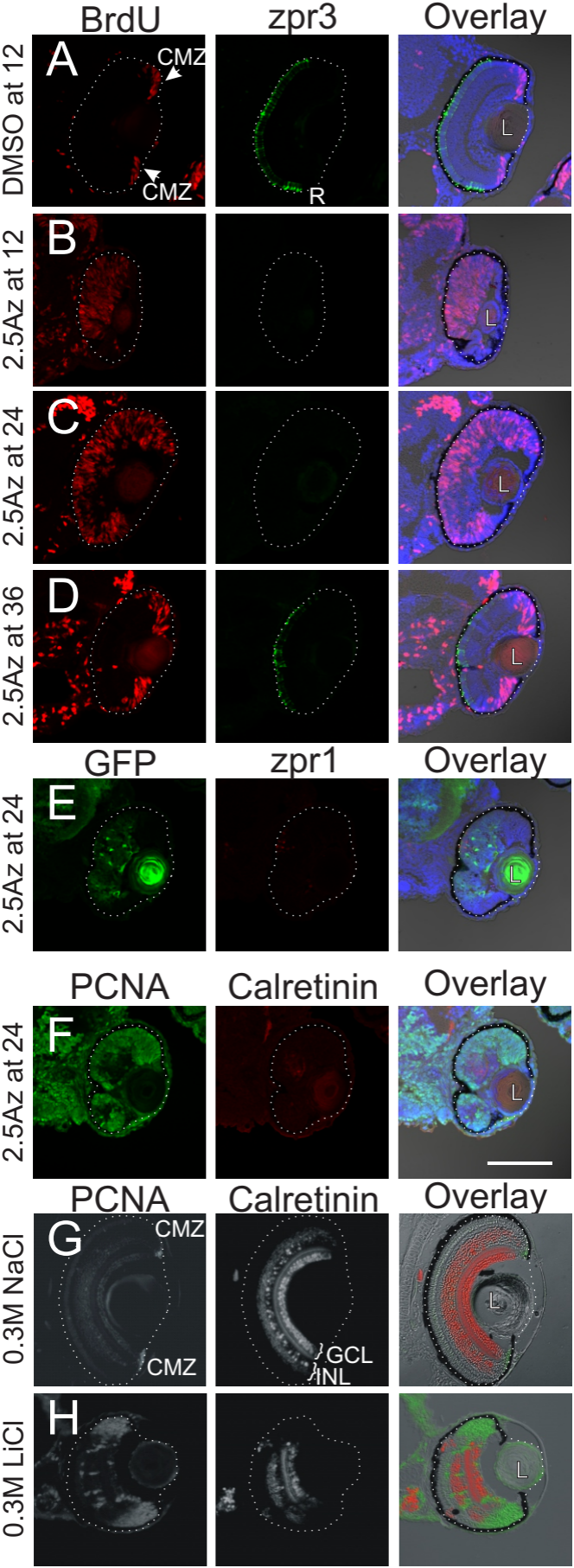

Supplement: Additional file 1 — Figure S1. Further evidence that inhibition of GSK3β blocks retinal differentiation and maintains proliferative progenitors. A pulse of BrdU provided 2 h prior to fixation at 72 hpf of fish treated with DMSO starting at 12 hpf shows the progenitors at the CMZ are actively moving through S-phase, while at the back of the retina zpr3 labels the rod photoreceptors (A). Treatment with 2.5 μM 1-azakenpaullone beginning at 12 hpf prevents rod differentiation and BrdU-positive cells are found throughout the retina (B). Treatment with 1-azakenpaullone beginning at 24 or 36 hpf allows some rods to differentiate, though there are still expanded pools of proliferating progenitors (C, D). Fish treated with 1-azakenpaullone beginning at 24 hpf also have expanded PCNA-positive domains, loss of calretinin-positive neurons, loss of GFP-positive Müller glia, and loss of zpr-1 labeled double cones (E, F). Inhibition of GSK3β with a 1 h treatment of 0.3 M LiCl at 48 hpf shows effects on retinal development similar to treatment with 1-azakenpaullone, with a reduced number of calretinin-positive neurons and an expanded CMZ (PCNA-labeled) compared with controls treated with 0.3 M NaCl (G,H). Scale bar. [file 1749-8104-7-30-S1.pdf]
